# Sleep duration, vegetable consumption and all-cause mortality among older adults in China: a 6-year prospective study

**DOI:** 10.1186/s12877-021-02278-8

**Published:** 2021-06-21

**Authors:** Chen Bai, Muqi Guo, Yao Yao, John S. Ji, Danan Gu, Yi Zeng

**Affiliations:** 1 School of Labor and Human Resources, Renmin University of China, Beijing, China; 2 Department of Global Health and Population, Harvard T.H. Chan School of Public Health, Harvard University, Boston, USA; 3 Center for Healthy Aging and Development Studies, National School of Development, Peking University, Beijing, China; 4 Environmental Research Center, Duke Kunshan University, Kunshan, Jiangsu China; 5 Independent Researcher, New York, NY 10017 USA; 6 Center for Healthy Aging and Development Studies, Raissun Institute for Advanced Studies, National School of Development, Peking University, No. 5 Yiheyuan Road, Beijing, 100871 Haidian District China; 7 Center for the Study of Aging and Human Development and Geriatrics Division, Medical School of Duke University, Durham, NC 27705 USA

**Keywords:** Sleep duration, Vegetables consumption, Mortality, Interaction, Older adults, Oldest-old, CLHLS

## Abstract

**Background:**

Sleep duration and vegetable consumption are associated with mortality at old age (termed as sleep-mortality linkage and vegetable-mortality linkage, respectively). Yet, little is known about the interplay of sleep duration and vegetable consumption on mortality.

**Methods:**

A dataset of nationwide longitudinal survey with 13,441 participants aged 65 years or older recruited in 2008 and followed up till 2014 was used. Sleep duration was classified into five groups (≤5, 6, 7–8, 9, and ≥ 10 h/day). Vegetable consumption was classified as either high frequency (eating vegetables almost daily) or low frequency. We used parametric Weibull hazard regression models to estimate associations of sleep duration and frequency of vegetable consumption with mortality, adjusting for demographics, socioeconomic factors, family/social support, health practice, and health conditions.

**Results:**

Over the six-year study period, when only demographics were present, participants sleeping ≤5, 6, 9, and ≥ 10 h/day had relative hazard (RH) of mortality 1.18 (*p* < 0.001), 1.14(*p* < 0.01), 1.06 (*p* > 0.1), and 1.30 (*p* < 0.001), respectively, compared to those sleeping 7–8 h/day. The HRs were attenuated to 1.08 (*p* < 0.05), 1.08 (*p* < 0.05), 1.09 (*p* < 0.1), 1.18(*p* < 0.001), respectively, when all other covariates were additionally adjusted for. High frequency of eating vegetables was associated with 22% lower risk of mortality (RH= 0.78, *p* < 0.001) compared to low frequency in the demographic model, and with 9% lower risk (RH = 0.91, *p* < 0.05) in the full model. Subpopulation and interaction analyses show that the sleeping-mortality linkage was stronger in female, urban, oldest-old (aged ≥80), and illiterate participants compared to their respective male, rural, young-old, and literate counterparts. High frequency of vegetable intakes could offset the higher mortality risk in participants with short-sleeping duration, but low frequency of eating vegetables could exacerbate mortality risk for participants with either short or long sleep duration; and except for few cases, these findings held in subpopulations.

**Conclusions:**

Too short and too long sleep durations were associated with higher mortality risk, and infrequent vegetable consumption could exacerbate the risk, although frequent vegetable intake could offset the risk for short sleep duration. The relationship between these two lifestyles and mortality was complex and varied among subpopulations.

**Supplementary Information:**

The online version contains supplementary material available at 10.1186/s12877-021-02278-8.

## Background

Sleep has been recommended as a crucial component to maintain a high quality of life and good health [1]. A growing body of epidemiological studies demonstrate that both long and short sleep durations are significant predictors for all-cause mortality among older adults [2–5]. There is plenty of evidence showing that the association of sleep duration with mortality might be partially modified by changes of dietary intakes [6]. Vegetable consumption has been verified as a strong predictor of both survival and healthy sleep patterns in older adults [7, 8]. For example, many studies have shown that a high frequency of vegetable consumption was linked to lower risk of mortality among older adults in Spain [9], Australia [10], and Japan [11]. Additionally, vegetable consumption also plays an important role in affecting sleep duration. Evidence shows that adherence to a Mediterranean diet, featured by daily consumption of vegetables and fruit, is associated with lower risk of changes in sleep duration and better sleep quality in older adults [12]. Some studies have further shown that low intakes of vegetables were associated with both longer and shorter sleep durations than recommended, and that lower fiber/carotenoids would lead to poor sleep quality and even increased risk of incident insomnia [13, 14].

Although associations between sleep duration, vegetable, consumption and mortality among older adults have been well documented in Western countries, it is still unknown how the interrelationship between sleep duration and vegetable consumption is linked with subsequent mortality risk among older adults in China, a country with the largest aging population in the world. As for the linkage between sleep duration and mortality, in addition to several subnational-level studies [15, 16], there are only handful studies using national representative samples which found that both short or long sleep durations were associated with higher mortality risk among older adults in China [17, 18].

There are numerous theoretical frameworks on the relationship between health behaviors and health outcomes. However, most theoretical frameworks only focused on single health behavior indicator and its related health outcomes. As any health outcome is influenced by a wide set of health behaviors [19], and as not all individuals respond in the same way to the same behavior [20], it is thus necessary to consider interactions between health behaviors in modeling health outcomes. According to the Compensatory Carry-Over Action Model (CCAM) proposed by Lippke [21], different health behaviors (i.e., physical activity and fruit/vegetable consumption) are interrelated, and they are driven by underlying mechanisms that promote a healthy lifestyle and prevent chronic diseases or mortality risk. In this context, considering the prior findings that vegetable consumption is either associated with lower mortality risk [22, 23] or with good quality of sleep among older adults [24], it is reasonable to postulate that the interplay between vegetable intake and sleep duration should be interactively associated with mortality risk among older populations.

In the present study, based on CCAM, we aim to explore the associations of sleep duration and vegetable consumption with mortality by using the data from the Chinese Longitudinal Healthy Longevity Survey (CLHLS), a nationally representative prospective cohort study of older adults in China. We also aim to investigate the interaction effect of vegetable consumption in association of sleep duration with mortality, to help draw proper prevention and intervention implications on healthy aging and longevity.

## Methods

### Sample

We used a nationally representative sample of 13,441 respondents from the 2008 wave of the Chinese Longitudinal Healthy Longevity Survey (CLHLS) and their follow-up waves in 2011 and 2014. The CLHLS began in 1998 focusing on the oldest-old aged 80 years or older and was carried out in a randomly selected half of the counties/cities from 22 out of 31 provinces in Mainland China (thereafter China), aiming to interview all centenarians in the sampled counties/cities. Comparable numbers of nonagenarians and octogenarians were recruited with predesignated age and sex based on the month of birth of local centenarians in the survey. Starting from 2002, the CLHLS recruited older adults aged 65–79 to reflect the entire older adult population of China with roughly every five individuals aged 65–79 per four centenarians in each local county/city. The sample was further expanded to include a county in Hainan Province in 2008, making a total of 23 provinces as its coverage. The sampled provinces covered nearly 90% of the total population according to the 2010 census. The CLHLS has the largest sample of centenarians, nonagenarians, and octogenarians in the contemporary world [25]. Well-trained professionals conducted face-to-face interviews with structured questionnaires containing comprehensive measurements of demographic and socioeconomic status, health behaviors [26]. Detailed protocols and study design of the CLHLS have been documented elsewhere [27]. The CLHLS interviewed 16,360 respondents aged 65 or older in 2008, of which 5,597 survived to the 2014 wave, 7,844 died before the 2014 wave, and 2,919 were lost to follow-up. Those lost to follow-up were excluded in the present study, leaving 13,441 participants in the final sample for analyses.

### Measurements

#### Sleep duration

Self-reported sleep duration (including both nighttime and daytime naps) was collected using the following question in the CLHLS 2008 questionnaire: “how many hours on average do you sleep every day, including daytime naps?” The self-reported sleep duration is commonly used in sleep studies [28–30] and its validity has been validated [31]. The sleep duration without distinguishing daytime naps and nightlight sleep is also commonly used in the literature [7, 32–34], although some studies reported different mechanisms and linkages with health [35–37]. For some respondents who were not able to report the duration, the next-of-kin answered it as a proxy response. A validation study of CLHLS data showed that there was no substantial bias by using the next-of-kin as proxies for objective and factual questions, such as sleep duration [38]. For the purpose of capturing a possible non-linear association between sleep duration and mortality, we followed previous studies and classified sleep hours into five categories (≤5, 6, 7–8, 9, and ≥ 10 h/ per day) [3, 39]. We used the category of 7–8 h as the reference category which was recommended as preferable sleep time by previous studies [3, 39].

#### Vegetable consumption

Vegetable consumption was measured by self-reported frequency of consuming fresh vegetables using a single overall question “how often do you have vegetables?” with options of “every day or almost every day,” “often,” “occasionally,” and “rarely or never.” The single general question is a common method for assessing the overall dietary habit in the field of dietetic studies [40, 41]. To facilitate the interpretation of the findings, we coded those who answered having such food “every day or almost every day” or “often” as “high frequency”, and coded those who answered “occasionally” or “rarely or never” as “low frequency”. Original ordinal categories were also tested, and results are more or less similar.

#### Mortality risk

Our primary outcome was all-cause mortality in the study period 2008–2014 with both survival status and the length of exposure to death as dependent variables in survival analysis. Survival status was identified as whether a respondent was dead or alive in the wave of 2014. Death information was collected from death certificates and confirmed by the next-of-kind. If death certificate was not available, a confirmation was obtained from local Residential Committee, Village Committee, or a community doctor. Data quality on mortality is relatively high [27].

#### Covariates

Our analyses controlled for a variety of confounders that were previously evidenced to link to sleep, vegetable consumption, and mortality [9–11, 18]. The factors included demographic, socioeconomic status (SES), family/social supports, health practices, and health conditions. Demographic variables included chronological age, sex, and urban/rural residence (urban vs. rural). SES were measured with four indicators including education (literate with at least 1 year formal education vs. illiterate with no years of schooling), economic independence (having a retirement wage/pension and/or own earnings vs.no), primary lifetime occupation (white collar occupation vs. other), and health care accessibility when in need (yes vs. no). Family/social supports were measured by current marital status (married vs. no), proximity to children (close vs. not close; defined as either having a co-resident child or having a child living in the same neighborhood). Health practices included smoking status (currently smoking yes vs. no) and physical exercise (currently participating regularly vs. not participating). Health conditions included physical functioning, cognitive functioning and self-reported health. Specifically, physical functioning was measured by disability in activities of daily living (ADL). The CLHLS assessed disability in ADL according to the Katz Index, including self-reported six tasks performed by individuals in daily life: eating, dressing, bathing, indoor transfer, toileting, and incontinence [42]. We defined a participant as ADL disabled if he or she needed assistance in performing any of those six items, and not disabled otherwise. Self-reported health was evaluated by the question “In general, would you say your health is: very good, good, fair, poor, or very poor?”. The responses were dichotomized into poor (fair/bad/very bad) health vs. good (very good/good) health. Cognitive function was assessed using a culturally adapted Chinese version of the Mini-Mental State Examination (MMSE) [38, 43]. We used the score of ≥24 out of total 30 as the cutoff value to distinguish normal cognition from impaired cognition [43].

### Statistical analysis

We first described the baseline characteristics of the study population classified by sleep duration categories (≤5 h, 6 h, 7–8 h, 9 h, ≥10 h). We then employed the Weibull hazard regression models to estimate the association between sleep duration, vegetable consumption and mortality [18, 44]. Five sequential models of Weibull hazard regressions were designed. Models I and II calculated relative hazards (RH) of mortality for sleep duration and vegetable consumption, respectively, controlling for demographics. Model III pooled Models I and II with additional controls for socioeconomic factors. Model IV further controlled for family/social supports, and health practices in Model III. Model V (i.e., full model) additionally controlled for health conditions. Whether the association between sleep duration and mortality varied by vegetable consumption, was also examined by using an interaction term of sleep duration and vegetable consumption. Finally, the non-interaction models and the interaction models were further stratified by gender, age, urban-rural residence, and education level group [45]. Except for sex, education, and primary lifetime occupation, all other variables were considered as time-varying covariates. The sampling weight was applied in the descriptive table only as an Appendix. For modeling, the sampling weight was not applied as it would introduce biases when the variables used for constructing the sample weight were already in analyses [46, 47]. This practice is consistent with many previous studies [18, 23, 24].

As there were losses to follow-up. Alternative analyses including those who were lost to follow-up by imputing their survival information only slightly altered the results. The imputed survival status was based on a multiple imputation method by assuming that the survival status and the length of survival would be the same if they had the same characteristics in demographics, SES, social connection, health practice, and health conditions at a previous wave as the respondents who were followed-up at a subsequent wave (see Appendix Tables A2 and A3).

All analyses were performed using STATA version 15.0 (Stata Corp LLC, College Station TX, USA).

## Results

Table 1 presents unweighted frequency distribution of characteristics of survey participants classified by sleep duration at the 2008 wave. About 36% of the interviewed participants used to sleep 7–8 h per day, the highest frequency of sleep duration, followed by those with sleeping 10h hours (nearly 30%), 6 h (13.6%), less than 5 h (11.8%), and 9 h (8.4%). Nearly 88% of the participants often eating vegetable. About 60% of the participants died 6 years later after interview in 2008. The weighted distributions were mildly different and presented in Appendix (see Table A1).

**Table 1:** Characteristics of study participants, classified by sleep duration (unweighted)

	Total	Hours of sleep per day					***p***-value
		≤5	6	7–8	9	≥10	
**# of participants**	13,441	1,588	1,826	4,877	1,133	4,017	NA
% participant across sleep hours	100.0	11.81	13.59	36.28	8.43	29.89	NA
**% Death in 2008–2014**	58.4	57.0	52.8	51.2	56.0	70.8	< 0.001
**Frequency of vegetable consumption**							
% High	87.6	83.6	88.5	89.4	89.8	86.4	< 0.001
**Demographics**							
Age (mean, years)	87.0	86.2	85.0	85.0	86.0	91.1	< 0.001
% Aged over 80 years	73.1	72.7	67.1	65.8	70.8	85.7	< 0.001
% Male	42.9	37.8	42.5	44.7	49.8	41.1	< 0.001
% Urban	36.7	34.8	39.3	37.4	35.7	35.8	0.027
**Socioeconomic factors**							
% 0 years of schooling	63.5	66.9	59.2	59.6	60.8	70.0	< 0.001
% Economic independence	25.8	25.1	31.1	30.7	26.0	17.8	< 0.001
% Professional occupation	9.4	8.2	10.5	10.9	7.9	8.0	< 0.001
% Access to healthcare	92.4	86.7	90.4	92.8	95.0	94.5	< 0.001
**Family/Social support**							
% Married	32.7	32.0	37.0	38.2	36.8	23.3	< 0.001
% Close proximity to children	87.3	84.3	86.9	87.1	88.1	88.7	< 0.001
**Health practice**							
% Doing regular exercise	27.3	28.1	30.2	30.7	28.9	28.3	< 0.001
% Currently smoking	17.9	16.9	19.4	18.6	18.8	16.5	0.016
**Health condition**							
% ADL disabled	20.6	21.5	18.4	14.3	13.0	31.2	< 0.001
% Cognitively impaired	44.3	42.2	39.9	36.5	36.9	58.6	< 0.001
% Self-reported poor health	51.1	67.9	59.1	47.9	44.2	46.6	< 0.001

Note: (1) *NA* not applicable. (2) Age was measured in mean, whereas all other variables were measured in percentage. (3) Except for the total sample whose unweighted proportion was calculated across sleep hours, unweighted percentages for all other variables were calculated within each sleep hour group. (4) Except for the variable death, all other variables were measured at baseline wave in 2008. (5) *p* values were based on chi-square tests (for categorical variables) except for mean age that was derived from analysis of variance

Table 2 presents the associations of vegetable consumption and sleep duration with mortality in term of relative hazards (RH) under different models controlling for different covariates. Model I shows that compared to normal sleep duration hours (7–8 h/day), sleeping ≤5 h/day, sleeping 6 h/day, and sleeping ≥10 h/day were associated with 18% (RH= 1.18, *p* < 0.001), 14% (RH=1.14, p<0.001), and 30% (RH = 1.30, p < 0.001) higher risk of mortality, respectively when only demographics were adjusted for. Model II shows that older adults who reported a high frequency of vegetable consumption had a 22% lower risk of mortality (RH = 0.78, *p* < 0.001) than those reporting a low frequency when only demographic factors were controlled for. Models III and IV reveal that the associations were only slightly altered when these two variables simultaneously present in modeling in addition to controlling for socioeconomic factors, family/social support, and health practice. When health conditions were further controlled for in Model V, the associations of sleep duration and vegetable consumption with mortality were mildly or moderately attenuated, yet they were still statistically significant. For example, RHs were reduced to 1.08 (*p* < 0.05) and 1.18 (*p* < 0.001) for sleep durations of ≤5 h/day and ≥ 10 h/day, respectively; and HR was reduced 0.91 (*p* < 0.05) for high frequency of vegetable.

**Table 2:** Relative hazards of subsequent mortality by sleep duration (per day) and vegetable consumption, CLHLS, the 2008–2014 panel

	Model I	Model II	Model III	Model IV	Model V
**Sleeping hours/day**					
≤ 5 h (7–8)	1.18***		1.15***	1.15***	1.08*
6 h (7–8)	1.14***		1.13**	1.12**	1.08*
9 h (7–8)	1.06		1.07	1.08	1.09+
≥ 10 h (7–8)	1.30***		1.29***	1.27***	1.18***
**Frequency of vegetable consumption**					
High (low)		0.78***	0.80***	0.82***	0.91*
**Covariates**					
***Demographics***					
Age (mean, years)	1.08***	1.08***	1.07***	1.07***	1.06***
Men (women)	1.24***	1.25***	1.34***	1.44***	1.52***
Urban (rural)	0.88***	0.89***	0.94*	0.97	0.93**
***Socioeconomic factors***					
1+ years of schooling (0)			0.95	0.97	1.02
Economic independence (no)			0.71***	0.77***	0.78***
Professional occupation (no)			1.09+	1.11*	1.07
Access to healthcare (no)			0.90**	0.91*	0.95
***Family/Social support***					
Currently married (no)				0.80***	0.81***
Close proximity to children (no)				1.09*	1.07*
***Health practice***					
Doing regular exercise (no)				0.92*	0.96
Currently smoking (no)				0.69***	0.78***
***Health condition***					
ADL disabled (no)					1.60***
Cognitively impaired (no)					1.47***
Self-reported good health (no)					1.16***
*N (observations)*	20,774	20,774	20,774	20,774	20,774
-log likelihood	15,763.3***	15,997.0***	15,681.2***	15,566.0***	15,209.9***

Note: Relative hazards were obtained from parametric survival analyses. The category of each variable in the parentheses is the reference group of that variable. ***p < 0.001, **p < 0.01, *p < 0.05, +*p* < 0.1

Panel A of Table 3 presents HRs of mortality for sleep duration and frequency of vegetable when they were simultaneously included in the full model as independent variables for the total sample and for different subgroups by sex (men and women), age (young older adults and oldest-old adults), urban/rural residence, and educational level (illiterate and literate). Panels B and C of Table 3 present the interaction between sleep duration and vegetable consumption in associating with subsequent mortality risk in the same full model and subpopulations as those in Panel A. Several findings care be summarized as follows.

**Table 3:** Relative hazards of mortality for the interaction between sleeping hours (per day) and vegetable consumption, CLHLS, the 2008–2014 panel

	Total	Women	Men	Young old	Oldest old	Rural	Urban	Illiterate	Literate
**Panel A**									
≤ 5 h and not often eating vegetables	1.08*	1.09+	1.08	0.89	1.09*	1.07	1.11	1.08+	1.07
6 h and not often eating vegetables	1.08*	1.11*	1.05	1.03	1.08*	1.06	1.14*	1.08	1.10
**7–8 h and not often eating vegetables**	1.00	1.00	1.00	1.00	1.00	1.00	1.00	1.00	1.00
9 h and not often eating vegetables	1.09+	1.19**	0.96	0.79	1.11*	1.10	1.06	1.13*	0.98
≥ 10 h and not often eating vegetables	1.18***	1.21***	1.13**	1.16	1.18***	1.15***	1.21***	1.19***	1.13*
High frequent intake of vegetables (low)	0.91**	0.96	0.82***	0.95	0.90**	0.96	0.83***	0.92*	0.85*
**Panel B**									
≤ 5 h and not often eating vegetables	1.33***	1.44**	1.19	1.00	1.33**	1.33**	1.32+	1.37**	1.15
6 h and not often eating vegetables	1.08	1.09	1.05	1.52	1.04	1.00	1.20	1.10	1.03
**7–8 h and not often eating vegetables**	**1.00**	**1.00**	**1.00**	**1.00**	**1.00**	**1.00**	**1.00**	**1.00**	**1.00**
9 h and not often eating vegetables	0.90	0.91	0.85	0.75	0.90	0.88	0.96	1.04	0.60+
≥ 10 h and not often eating vegetables	1.33***	1.42***	1.20	1.27	1.34***	1.23*	1.46**	1.32**	1.30+
≤ 5 h and often eating vegetables	1.02	1.10	0.89	0.94	1.02	1.02	1.02	1.03	0.95
6 h and often eating vegetables	1.07	1.21*	0.89	1.05	1.08	1.07	1.07	1.09	1.00
7–8 h and often eating vegetables	0.99	1.09	0.85+	1.07	0.98	1.01	0.95	1.01	0.90
9 h and not eating vegetables	1.09	1.33**	0.83+	0.85	1.12	1.14	1.02	1.15+	0.93
≥ 10 h and often eating vegetables	1.13*	1.27**	0.95	1.24	1.13*	1.16+	1.11	1.18*	0.98
**Panel C**									
≤ 5 h and not often eating vegetables	1.34***	1.32**	1.40**	0.94	1.35**	1.31**	1.38**	1.36**	1.29+
6 h and not often eating vegetables	1.10	1.02	1.24	1.42	1.06	0.99	1.27+	1.09	1.15
7–8 h and not often eating vegetables	1.01	0.92	1.18+	0.93	1.02	1.00	1.05	0.99	1.12
9 h and not often eating vegetables	0.91	0.84	1.00	0.70	0.92	0.87	1.00	1.02	0.66
≥ 10 h and not often eating vegetables	1.35***	1.30***	1.42***	1.18	1.36***	1.22*	1.53***	1.29***	1.45*
≤ 5 h and often eating vegetables	1.03	1.01	1.06	0.88	1.03	1.00	1.07	1.02	1.06
6 h and often eating vegetables	1.08+	1.11+	1.05	0.98	1.09*	1.06	1.13+	1.07	1.11
**7–8 h and often eating vegetables**	**1.00**	**1.00**	**1.00**	**1.00**	**1.00**	**1.00**	**1.00**	**1.00**	**1.00**
9 h and not eating vegetables	1.11*	1.22**	0.98	0.79	1.14*	1.13+	1.07	1.13*	1.04
≥ 10 h and often eating vegetables	1.15***	1.17***	1.12*	1.15	1.15***	1.14**	1.17**	1.17*	1.10
N (observations)	20,774	11,693	9081	6003	14,771	11,984	8790	13,017	7757

Note: Relative hazards were obtained from parametric survival analyses. The reference group in Panel B is “7–8 h and not often eating vegetables”, whereas the reference group in Panel C is “7–8 h and often eating vegetables.” All models controlled for all covariates in Table 1. Relative hazards for all covariates were not presented. ***p < 0.001, **p < 0.01, *p < 0.05, +*p* < 0.1

First, the results without interaction (Panel A) indicate that the association between sleep duration and mortality was stronger in women, oldest-old adults, urban older adults, and illiterate older adults than their respective counterpart men, young older adults, rural older adults, and illiterate older adults.

Second, there is a double jeopardy for too short and too long sleep durations if they were combined with infrequent intake of vegetables. For example, among the participants with low frequency of vegetable consumption, those with ≤5 h/day or with ≥10 h/day had a 33–35% more risk of mortality compared to those who used to sleep 7–8 h/day with either frequent or infrequent intake of vegetables. The increased RHs were 1.08 for too short sleep (≤ 5 h/day) and 1.18 for too long sleep (≥10 h/day) when the interaction of these two factors was considered. Such double jeopardy was also valid in subpopulations except among young older adults.

Third, among those with short sleep duration (≤ 5 h/day), their higher mortality risk could be diminished by frequent intake of vegetables. This is universal across subpopulations except among young older adults. However, among those with long sleep duration (≥10 h/day), the higher mortality risk persisted if they used to frequently eat vegetables, although the higher mortality risk seemed slightly lower compared to the case when the interaction term was not taken into account. These findings imply that eating more vegetables could benefit more for those with short sleep hours.

Fourth, among those sleeping 7–8 h/day, the frequency of intake of vegetables was not associated with mortality. This indicates that whether often having vegetables or not-often having vegetables had no impact on mortality risk of older adults as long as they used to sleep 7–8 h daily. In other words, eating more vegetables or eating less vegetables would not reduce or increase mortality risk among older adults with sleeping 7–8 h per day.

## Discussion

To our knowledge, this is the first study to explore associations of sleep duration, vegetable consumption, and their interactions on all-cause mortality in a large population-based perspective cohort study of Chinese older adults. The results reveal that both short (≤5 h/day) and long (≥10 h/day) sleep durations were associated with increased risks of mortality, compared to 7–8 h/day. We also found that the high frequency of vegetable consumption was associated with substantially lower mortality risk. These associations were stronger in female, oldest-old, urban, and illiterate participants. Moreover, a significant multiplicative interaction between vegetable consumption and sleep duration on mortality risk was detected. A low frequency of vegetable consumption combined with short or long sleep hours could have a double jeopardy of mortality, although a high frequency of vegetable intake could reduce mortality risk among participants with short sleep duration. The findings are valid in all subgroups except for ages 65–79 in which both the sleeping duration and the frequency of vegetable consumptions were not statistically associated with mortality. These findings well support that health behavioral factors interact each other to jointly influence health outcomes as reflected in CCAM [21].

Our finding is consistent to the existing literature [48–53]. For instance, a meta-analysis found globally adverse effects of a long sleep duration on mortality and moderately adverse effects of a short sleep duration only on mortality in North American populations [2]. Several other studies on Chinese middle-aged or older adults in Hong Kong [51] and Shanghai [4] found that a prolonged rather than a shorter sleep duration was more significantly associated with increased mortality even controlling for participants’ health status. One study using the 2005 and 2008 waves of the CLHLS confirmed such pattern in older women and at oldest-old ages [18]. Besides, several meta-analyses provided solid evidence that a higher consumption of vegetables is associated with a lower risk of all-cause mortality [54, 55]. Some empirical studies also confirmed that vegetable consumption was inversely associated with risk of all-cause mortality among older adults in China [22, 23]. Although the interaction effect of vegetable consumption with sleep hours revealed by our study on mortality has not been empirically reported in the existing literature, it partially coincided with many prior studies on the directional associations of vegetable consumption to sleep duration or to mortality, respectively [9–14, 22, 23, 54, 55]. The existing literature has also documented that higher prevalence of mortality risk linked to sleep disorders or a low vegetable consumption was more likely to be found among disadvantaged older adults, such as women, the oldest-old, the less educated and so on due to their low socioeconomic status and poor health status [56].

Several mechanisms have been suggested to explain the associations among sleep durations, vegetable consumption, and mortality. An adequate sleep duration is a basic guarantee for normal immune function. However, as for a long sleep duration, some studies demonstrated that a long sleep duration has been associated with fatigue, lower immune function, changing in cytokine levels, depression, underlying disease process or physiological reduction of the photoperiod, which could lead to increased mortality [4, 57]. As for a short sleep duration, insufficient sleep hours could damage the immune system, which is more likely to cause body dysfunctions [58] and increase risk for chronic health conditions [33, 59], such as hypertension [60], cardiovascular disease [61], type 2 diabetes [62], and obesity [63]. Besides, limited sleep hours may also gradually change certain neuroendocrine systems and sensitize individuals to stress-related disorders, like depression [64]. All these adverse health outcomes are main risk factors for mortality among older adults. Vegetable is an important source of micro-nutrients with antioxidant, polyphenols, carotenoids, vitamin C, fiber, potassium, flavonoids, and other and other biologically active properties [22, 23], which are proved to act synergistically through numerous biological mechanisms to prevent against a wide range of chronic diseases and premature mortality [13, 14]. For instance, vegetable intakes have been shown to reduce blood pressure, cholesterol levels, inflammation and to stimulate vascular and immune functioning [65]. Vegetables may also have a beneficial effect on modulating steroid hormone metabolism and concentrations. Additionally, antioxidants in vegetables may help to neutralize reactive oxygen species [66].

Though mechanism underlying the association between interaction effect of sleep duration and vegetable consumption on mortality risk remains unclear, some potential pathways can be suggested. First, since both an extreme sleep duration and a low frequency of vegetable consumption would increase risk of mortality respectively, it is reasonable to infer that interaction of an extreme sleep duration and a low frequency of vegetable could trigger a double jeopardy of mortality. Second, some mechanisms have been proposed of the reciprocal association between sleep disruption and vegetable intake that may subsequently lead to reverse health outcomes or even mortality. On one hand, laboratory studies have suggested that disrupted sleep would change appetite-related hormones ghrelin and leptin, which may increase the preference for energy-dense foods probably leading to lower consumption of vegetables [67]. Some experimental studies also found that both short and long sleep duration may impair energy homeostasis through unhealthy dietary patterns, causing lower vegetable intake [68, 69]. On the other hand, sleep serves as an important role in protecting the human body against the harmful impacts of free radicals caused by a high metabolic rate during waking times [66]. It was previously suggested that metabolic rate during sleeping time appears much lower than awake time, when providing an opportunity to re-generate the enzymes influenced by free radical [70]. In this regard, vegetables with antioxidant components are expected to maintain the regular sleep-wake cycles by improving mitochondrial function and energy metabolism through decreasing protein content, which may lead to a reduction in the synthesis of brain sleep inductors and sleep parameters [71].

Third, mortality protective benefits of frequent vegetable intake may mitigate mortality risk brought by short sleep duration. As noted in the literature, short sleepers are more likely to take energy- and fat-dense food [72]. Saturated fat intake is associated with risk of increased all-cause mortality and risk of mortality from atherosclerotic vascular disease [73]. Frequent vegetable intake among short sleepers may reduce their fat intake and mitigate the burden of fat on mortality risk. Some studies found that a combination of high fruit and vegetable and low saturated fat intakes was protective against mortality, while consuming either low saturated fat or high fruit and vegetables alone was not associated with lower mortality risk, compared to those having neither saturated fat nor fruit and vegetables [74]. Adherence to frequent vegetable intake or greater vegetable intake may help adjust extreme sleep durations to the recommended level [75] and improve sleep quality without changing sleep duration [76, 77].

Another interesting finding is that young-old adults appear to be escapers from risk or double jeopardy of mortality by extreme sleep hours and/or a low frequency of vegetable intake. The lack of association between sleep duration or frequency of vegetable consumption among young-old adults is somewhat consistent with previous studies. For example, a prior Chinese study showed short sleep was not associated with mortality among young-old adults aged 65–79 [18]. It is possible that dramatic amplitude reduction and increase in frequency of delta waves, which are closely linked to sleep duration and its subsequent reverse health outcomes, are more likely among the oldest-old, rather among young-old adults [18]. Another Swedish study found that the sleep duration was not associated with 13-year mortality among adults aged 65 and over [78]. One recent meta-analysis further showed that shorter sleepers (groups with less than 7 h/day) were not associated with mortality compared to those with 7 h/day in 40 studies [79]. One Japanese study revealed that mortality risk was not statistically different among young-old adults aged less than 75 years between those who had a healthy diet (mainly composed of vegetables, seaweed, fish) and those who had a greasy diet (mainly composed of meat and fat) [65]. Young-old adults may still maintain some biological advantage compared to oldest-old adults that enable them to be immune from threats of short sleep and low frequency vegetables intake. Nevertheless, the reasons of lack of association among young-old adults are unclear, and more research is warranted to shed light on it.

This study bears the following limitations. First, it relied on self-reported sleep duration, which may have measurement errors. However, it is challenging and expensive to assess sleep duration by using actigraphy for thousands of very old participants from a large epidemiological survey. Furthermore, daytime naps were not separately modeled from the nocturnal sleep duration due to unavailability of data in the CLHLS. Although some studies suggested their similar associations with certain health outcomes [80], some other studies suggested their dissimilarity in linking with different health outcomes [35–37]. Future studies distinguishing these two types of sleep are clearly warranted. Similarly, our assessment of vegetable consumption was mainly based on the self-reported information on frequency intake of vegetables with a single overall question. No detailed dietary consumption information was collected including the amount/type of vegetables, which may limit the generalizability of our results. Applications using a structural questionnaire to collect more detailed dietary information is certainly needed to better reflect the individual dietary pattern.

Second, sleep quality was not modeled. There is plenty of evidence showing that sleep quality is strongly linked with mortality [18], which may confound the linkages between mortality and sleep duration and dietary pattern. Thus, the present study examined very basic sleeping and dietary patterns among Chinese older adults. Tea- or coffee- drinking behaviors and their timing could affect sleep patterns. Future research should conduct standard methods to capture more informative patterns of dietary and sleeping behaviors. Relatedly, in the present study, we only examined the interaction effect between sleep duration and vegetable intake on mortality. Future research is needed to disentangle the moderate role of vegetable consumption in the linkage between sleep duration and mortality and the moderate role of sleep duration in the linkage between vegetable intake and mortality. Third, the study has several unmeasured confounders including sleep related disorders (e.g. insomnia), sleep medication use, and other underlying diseases may also affect both sleep duration and mortality. However, prior studies found that even after controlling for insomnia, daily napping, sleep apnea, use of sleep medications and many other confounders, an extreme sleep duration was still associated with increased all-cause mortality [81]. Thus, the biases of our present study would not be substantial.

Despite limitations, our study made contribution to the existing literature. Our study extended the findings of sleep duration effect on mortality to adults at very old groups aged over 80 years with a focus on the interaction between vegetable intakes and sleep durations, despite most of relevant studies focusing on younger age groups. We examined the interactions among various subpopulations and found that the effects differed across the subpopulations and more pronounced among more vulnerable populations such as women, the oldest-old, and the illiterate. These findings could have important implications for implementing person-centered intervention programs and policies among Chinese older adults for achieving healthy aging. First, families and public health practitioners should pay attention to the potential adverse prognosis of extreme sleep durations and low vegetable intakes associated with mortality risk among older adults, particularly among the vulnerable groups of women, the oldest-old, and the illiterate. Second, given higher mortality risk among older adults living in rural areas who have extreme sleep durations and a low vegetable consumption, our finding may have important implications for promoting rural residents’ sleep and diet habits to mitigate mortality risk and reduce rural-urban health disparity, which is one objective of the newly launched nationwide project on Rural Revitalization guided by the Chinese Central Government [82]. Third, our findings also have some implications for future academic research. According to the CCAM model, instead of simply focusing on regulating older adults’ sleeping behaviors directly, more comprehensive and indirect intervention strategies should be designed, such as dietary therapies (e.g., high fiber diet) to reduce sleep-related mortality risk. Furthermore, further research should disentangle the interaction effect of sleep during and vegetable intake on mortality so that more specific recommendations to implement preventative and therapeutic strategies. In addition, it would be interesting to investigate possible biological and psychosocial mechanisms underlying the linkage between the interaction of sleep and vegetable consumption on mortality among older adults. Findings on the difference in the interactions among subpopulations also suggest a stratification of analysis.

## Conclusion

This 6-year follow-up prospective cohort study showed that associations between sleep duration and all-cause mortality among Chinese older adults turned out to be independent, net of a wide set of major covariates, and that the magnitude of the association was stronger for longer sleep durations than the shorter ones. Additionally, a significant interaction role of a high frequency of vegetable consumption in buffering negative effects of excessive sleep durations was found among women, the oldest-old, or the illiterate. These findings suggest that vegetable consumption can be a protective factor helping to alleviate the adverse effect of abnormal sleep durations on mortality risk.

## Supplementary Information

**Additional file 1.**

## Data Availability

The datasets generated and analyzed during the current study are available in the Peking University Open Research Data repository (https://opendata.pku.edu.cn/dataverse/CHADS). The datasets are also available at https://www.icpsr.umich.edu/web/NACDA/series/487.

## Abbreviation

ADL: Activities of daily living
CCAM: Compensatory Carry-Over Action Model
CLHLS: Chinese Longitudinal Healthy Longevity Survey
MMSE: Mini-Mental State Examination

## References

[1] Reid KJ, Martinovich Z, Finkel S, Statsinger J, Golden R, Harter K, et al. Sleep: a marker of physical and mental health in the elderly. Am J Geriatric Psychiatry. 2006;14(10):860–6. 10.1097/01.JGP.0000206164.56404.ba. 10.1097/01.JGP.0000206164.56404.ba 17001025

[2] Cappuccio FP, D'Elia L, Strazzullo P, Miller MA (2010). Sleep duration and all-cause mortality: a systematic review and meta-analysis of prospective studies. Sleep..

[3] Silva AA, de Mello RGB, Schaan CW, Fuchs FD, Redline S, Fuchs SC (2016). Sleep duration and mortality in the older adults: a systematic review with meta-analysis. BMJ Open.

[4] Cai H, Shu X, Xiang Y, Yang G, Li H, Ji B (2015). Sleep duration and mortality: a prospective study of 113,138 middle-aged and older adults Chinese men and women. Sleep..

[5] Kurina LM, McClintock MK, Chen J, Waite LJ, Thisted RA, Lauderdale DS (2013). Sleep duration and all-cause mortality: a critical review of measurement and associations. Ann Epidemiol.

[6] Huang Y, Wahlqvist ML, Lee M (2013). Sleep quality in the survival of elderly Taiwanese: roles for dietary diversity and pyridoxine in men and women. J Am Coll Nutr.

[7] Kim S, DeRoo LA, Sandler DP (2011). Eating patterns and nutritional characteristics associated with sleep duration. Public Health Nutr.

[8] Trichopoulou A, Kouris-Blazos A, Wahlqvist ML, Gnardellis C, Lagiou P, Polychronopoulos E, et al. Diet and overall survival in older adults people. BMJ. 1995;311(7018):1457–60. 10.1136/bmj.311.7018.1457. 10.1136/bmj.311.7018.1457 PMC2543726 8520331

[9] Olaya B, Moneta MV, Lara E, Miret M, Martín-María N, Moreno-Agostino D, et al. Fruit and vegetable consumption and potential moderators associated with all-cause mortality in a representative sample of Spanish older adults. Nutrients. 2019;11(8):1794. 10.3390/nu11081794. 10.3390/nu11081794 PMC6723290 31382535

[10] Nguyen B, Bauman A, Gale J, Banks E, Kritharides L, Ding D (2016). Fruit and vegetable consumption and all-cause mortality: evidence from a large Australian cohort study. Int J Behav Nutr Phys Act.

[11] Nagura J, Iso H, Watanabe Y, Maruyama K, Date C, Toyoshima H, et al. Fruit, vegetable and bean intake and mortality from cardiovascular disease among Japanese men and women: the JACC study. Br J Nutr. 2009;102(2):285–92. 10.1017/S0007114508143586. 10.1017/S0007114508143586 19138438

[12] Campanini MZ, Guallar-Castillón P, Rodríguez-Artalejo F, Lopez-Garcia E (2017). Mediterranean Diet and Changes in Sleep Duration and Indicators of Sleep Quality in Older Adults. Sleep.

[13] Noorwali EA, Cade JE, Burley VJ, Hardie LJ (2018). The relationship between sleep duration and fruit/vegetable intakes in UK adults: a cross-sectional study from the National Diet and nutrition survey. BMJ Open.

[14] Noorwali E, Hardie L, Cade J (2018). Fruit and Vegetable Consumption and Their Polyphenol Content Are Inversely Associated with Sleep Duration: Prospective Associations from the UK Women's Cohort Study. Nutrients.

[15] Liu X, Liu L. Sleep habits and insomnia in a sample of elderly persons in China. Sleep. 2005:224. 16408418

[16] Sun X, Ma T, Yao S, Chen Z, Xu W, Jiang X (2020). Associations of sleep quality and sleep duration with frailty and pre-frailty in an elderly population Rugao longevity and ageing study. BMC Geriatr.

[17] Lan T, Lan T, Wen C, Lin Y, Chuang Y (2007). Nighttime sleep, Chinese afternoon nap, and mortality in the elderly. Sleep..

[18] Qiu L, Sautter J, Liu Y, Gu D (2011). Age and gender differences in linkages of sleep with subsequent mortality and health among very old Chinese. Sleep Med.

[19] Umberson D, Karas Montez J. Social relationships and health: A flashpoint for health policy. J Health Soc Behav, 2010; 51(1_suppl): S54–S66. doi:10.1177/0022146510383501 10.1177/0022146510383501 PMC3150158 20943583

[20] Short SE, Mollborn S (2015). Social determinants and health behaviors: conceptual frames and empirical advances. Curr Opin Psychol.

[21] Lippke S (2014). Modelling and supporting complex behavior change related to obesity and diabetes prevention and management with the compensatory carry-over action model. J Diab Obes.

[22] Lo Y, Chang Y, Wahlqvist ML, Huang H, Lee M (2012). Spending on vegetable and fruit consumption could reduce all-cause mortality among older adults. Nutr J.

[23] Shi Z, Zhang T, Byles J, Martin S, Avery JC, Taylor AW (2015). Food habits, lifestyle factors and mortality among oldest old Chinese: the Chinese longitudinal healthy longevity survey (CLHLS). Nutrients..

[24] Lee Y, Chang Y, Lee Y, Shelley M, Liu C (2018). Dietary patterns with fresh fruits and vegetables consumption and quality of sleep among older adults in mainland China. Sleep Biol Rhythms.

[25] Zeng Y, Feng Q, Gu D, Vaupel JW. Demographics, phenotypic health characteristics and genetic analysis of centenarians in China. Mechanisms Ageing Dev. 2017; 165(Pt B): 86–97. 10.1016/j.mad.2016.12.010 PMC5489367 28040447

[26] Gu D. General data quality assessment of the CLHLS. In: Yi Z, Poston DL, Vlosky DA, Gu D, editors. Healthy longevity in China: demographic, socioeconomic, and psychological dimensions. Dordrecht: Springer Netherlands; 2008. p. 39–60. 10.1007/978-1-4020-6752-5_3.

[27] Gu D, Feng Q, Chen H, Zeng Y (2020). Chinese longitudinal healthy longevity survey. In D Gum Dupre ME. (eds.) Encyclopedia of gerontology and population aging. Cham: Springer. 10.1007/978-3-319-69892-2_968-1.

[28] Essien SK, Feng CX, Sun W, Farag M, Li L, Gao Y (2018). Sleep duration and sleep disturbances in association with falls among the middle-aged and older adults in China: a population-based nationwide study. BMC Geriatr.

[29] Lo JC, Groeger JA, Cheng GH, Dijk D, Chee MWL (2016). Self-reported sleep duration and cognitive performance in older adults: a systematic review and meta-analysis. Sleep Med.

[30] Nunes J, Jean-Louis G, Zizi F, Casimir GJ, von Gizycki H, Brown CD, et al. Sleep duration among black and white Americans: results of the National Health Interview Survey. J Natl Med Assoc. 2008;100(3):317–22. 10.1016/S0027-9684(15)31244-X. 10.1016/s0027-9684(15)31244-x 18390025

[31] Aili K, Åström-Paulsson S, Stoetzer U, Svartengren M, Hillert L (2017). Reliability of actigraphy and subjective sleep measurements in adults: the design of sleep assessments. J Clin Sleep Med.

[32] Patterson RE, Emond JA, Natarajan L, Wesseling-Perry K, Kolonel LN, Jardack P, et al. Short sleep duration is associated with higher energy intake and expenditure among African-American and non-Hispanic White adults. J Nutr. 2014;144(4):461–6. 10.3945/jn.113.186890. 10.3945/jn.113.186890 PMC3952622 24523490

[33] Magee CA, Holliday EG, Attia J, Kritharides L, Banks E (2013). Investigation of the relationship between sleep duration, all-cause mortality, and preexisting disease. Sleep Med.

[34] Štefan L, Radman I, Podnar H, Vrgoč G (2018). Sleep duration and sleep quality associated with dietary index in free-living very old adults. Nutrients.

[35] Jackson CL, Patel SR, Jackson WB, Lutsey PL, Redline S (2018). Agreement between self-reported and objectively measured sleep duration among white, black, Hispanic, and Chinese adults in the United States: multi-ethnic study of atherosclerosis. Sleep.

[36] Owusu JT, Wennberg AMV, Holingue CB, Tzuang M, Abeson KD, Spira AP (2019). Napping characteristics and cognitive performance in older adults. Int J Geriatr Psychiatr.

[37] Zonoozi S, Ramsay SE, Papacosta O, Lennon L, Ellins EA, Halcox JPJ, et al. Self-reported sleep duration and napping, cardiac risk factors and markers of subclinical vascular disease: cross-sectional study in older men. BMJ Open. 2017;7(6):e016396. 10.1136/bmjopen-2017-016396. 10.1136/bmjopen-2017-016396 PMC5726087 28674146

[38] Zeng Y, Poston DL, Vlosky DA, Gu D (2008). Healthy Longevity in China: Demographic, Socioeconomic, and Psychological Dimensions.

[39] Hirshkowitz M, Whiton K, Albert SM, Alessi C, Bruni O, DonCarlos L, et al. National sleep foundation’s sleep time duration recommendations: methodology and results summary. Sleep Health. 2015;1(1):40–3. 10.1016/j.sleh.2014.12.010. 10.1016/j.sleh.2014.12.010 29073412

[40] Mello Rodrigues V, Bray J, Fernandes AC, Luci Bernardo G, Hartwell H, Secchi Martinelli S, et al. Vegetable consumption and factors associated with increased intake among college students: a scoping review of the last 10 years. Nutrients. 2019;11(7):1634. 10.3390/nu11071634. 10.3390/nu11071634 PMC6682864 31319573

[41] Stea TH, Nordheim O, Bere E, Stornes P, Eikemo TA (2020). Fruit and vegetable consumption in Europe according to gender, educational attainment and regional affiliation—a crosssectional study in 21 European countries. PLoS One.

[42] Katz S, Ford AB, Moskowitz RW, Jackson BA, Jaffe MW (1963). Studies of illness in the aged. The index of ADL: a standardized measure of biological and psychosocial function. JAMA.

[43] An R, Liu GG (2016). Cognitive impairment and mortality among the oldest-old Chinese. Int J Geriatr Psychiatr.

[44] Luo Y, Zhang Z, Gu D (2015). Education and mortality among older adults in China. Soc Sci Med.

[45] Stata Bookstore: An introduction to survival analysis using Stata, Revised Third Edition. https://www.stata.com/bookstore/survival-analysis-stata-introduction/. Accessed 11 Apr 2020.

[46] Solon G, Haider SJ, Wooldridge JM (2015). What are we weighting for?. J Hum Resour.

[47] Winship C, Radbill L (1994). Sampling weights and regression analysis. Sociol Methods Res.

[48] Castro-Costa É, Me D, Ferri CP, Uchôa E, Joa F, Rocha FL (2011). Association between sleep duration and all-cause mortality in old age: 9-year follow-up of the Bambuí cohort study, Brazil. J Sleep Res.

[49] Cohen-Mansfield J, Perach R (2012). Sleep duration, nap habits, and mortality in older persons. Sleep..

[50] Mesas AE, López-García E, León-Muñoz LM, Guallar-Castillón P, Rodríguez-Artalejo F (2010). Sleep duration and mortality according to health status in older adults. J Am Geriatr Soc.

[51] Lee JSW, Auyeung TW, Leung J, Chan D, Kwok T, Woo J, et al. Long sleep duration is associated with higher mortality in older people independent of frailty: a 5-year cohort study. J Am Med Dir Assoc. 2014;15(9):649–54. 10.1016/j.jamda.2014.05.006. 10.1016/j.jamda.2014.05.006 24973244

[52] Ikehara S, Iso H, Date C, Kikuchi S, Watanabe Y, Wada Y, et al. Association of Sleep Duration with mortality from cardiovascular disease and other causes for Japanese men and women: the JACC study. Sleep. 2009;32(3):295–301. 10.1093/sleep/32.3.295. 10.1093/sleep/32.3.295 PMC2647783 19294949

[53] Patel SR, Ayas NT, Malhotra MR, White DP, Schernhammer ES, Speizer FE, et al. A prospective study of sleep duration and mortality risk in women. Sleep. 2004;27(3):440–4.39. 10.1093/sleep/27.3.440. 10.1093/sleep/27.3.440 15164896

[54] Aune D, Giovannucci E, Boffetta P, Fadnes LT, Keum N, Norat T, et al. Fruit and vegetable intake and the risk of cardiovascular disease, total cancer and all-cause mortality-a systematic review and dose-response meta-analysis of prospective studies. Int J Epidemiol. 2017;46(3):1029–56. 10.1093/ije/dyw319. 10.1093/ije/dyw319 PMC5837313 28338764

[55] Wang X, Ouyang Y, Liu J, Zhu M, Zhao G, Bao W, et al. Fruit and vegetable consumption and mortality from all causes, cardiovascular disease, and cancer: systematic review and dose-response meta-analysis of prospective cohort studies. BMJ. 2014;349(3):g4490. 10.1136/bmj.g4490. 10.1136/bmj.g4490 PMC4115152 25073782

[56] Gu D, Sautter J, Pipkin R, Zeng Y (2010). Sociodemographic and health correlates of sleep quality and duration among very old Chinese. Sleep..

[57] Grandner MA, Drummond SPA (2007). Who are the long sleepers? Towards an understanding of the mortality relationship. Sleep Med Rev.

[58] Besedovsky L, Lange T, Born J (2011). Sleep and immune function. Pflugers Arch.

[59] Kripke DF, Garfinkel L, Wingard DL, Klauber MR, Marler MR (2002). Mortality associated with sleep duration and insomnia. Arch Gen Psychiatry.

[60] Guo J, Fei Y, Li J, Zhang L, Luo Q, Chen G (2016). Gender- and age-specific associations between sleep duration and prevalent hypertension in middle-aged and elderly Chinese: a cross-sectional study from CHARLS 2011–2012. BMJ Open.

[61] Suzuki E, Yorifuji T, Ueshima K, Takao S, Sugiyama M, Ohta T, et al. Sleep duration, sleep quality and cardiovascular disease mortality among the elderly: a population-based cohort study. Prev Med. 2009;49(2):135–41. 10.1016/j.ypmed.2009.06.016. 10.1016/j.ypmed.2009.06.016 19573557

[62] Zhang S, Xie L, Yu H, Zhang W, Qian B (2019). Association between nighttime-daytime sleep patterns and chronic diseases in Chinese elderly population: a community-based cross-sectional study. BMC Geriatr.

[63] Lee PMY, Tse LA (2019). Association between sleep duration, bed time and obesity in community-dwelling Hong Kong Chinese elderly: a population-based study. Sleep Med.

[64] Meerlo P, Sgoifo A, Suchecki D. Restricted and disrupted sleep: Effects on autonomic function, neuroendocrine stress systems and stress responsivity. Sleep Med Rev. 2007, 2008;12(3):197–210. 10.1016/j.smrv.2007.07.007 18222099

[65] Iimuro S, Yoshimura Y, Umegaki H, Sakurai T, Araki A, Ohashi Y, et al. Dietary pattern and mortality in Japanese elderly patients with type 2 diabetes mellitus: does a vegetable- and fish-rich diet improve mortality? An explanatory study. Geriatr Gerontol Int. 2012;12:59–67. 10.1111/j.1447-0594.2011.00813.x. 10.1111/j.1447-0594.2011.00813.x 22435941

[66] Anderson JW, Baird P, Davis RH (2009). Health benefits of dietary fibre. Nutr Rev.

[67] Noorwali E, Hardie L, Cade J (2019). Bridging the reciprocal gap between sleep and fruit and vegetable consumption: a review of the evidence, potential mechanisms, implications, and directions for future work. Nutrients..

[68] Tan X, Chapman CD, Cedernaes J, Benedict C (2018). Association between long sleep duration and increased risk of obesity and type 2 diabetes: a review of possible mechanisms. Sleep Med Rev.

[69] Chapman CD, Nilsson EK, Nilsson VC, Cedernaes J, Rangtell FH (2013). Acute sleep deprivation increases food purchasing in men. Obesity.

[70] Wurtman RJ, Wurtman JJ, Regan MM, McDermott JM, Tsay RH, Breu JJ (2003). Effects of normal meals rich in carbohydrates or proteins on plasma tryptophan and tyrosine ratios. Am J Clin Nutr.

[71] Lana A, Struijk EA, Arias-Fernandez L, Graciani A, Mesas AE, Rodriguez-Artalejo F, et al. Habitual meat consumption and changes in sleep duration and quality in older adults. Aging Dis. 2019;10(2):267–77. 10.14336/AD.2018.0503. 10.14336/AD.2018.0503 PMC6457059 31011478

[72] Dashti HS, Scheer FAJL, Jacques PF, Lamon-Fava S, Ordovás JM (2015). Short sleep duration and dietary intake: epidemiologic evidence, mechanisms, and health implications. Adv Nutr.

[73] Kromhout D, Bloemberg B, Feskens E, Menotti A, Nissinen A (2000). Saturated fat, vitamin C and smoking predict long-term population all-cause mortality rates in the seven countries study. Int J Epidemiol.

[74] Tucker KL, Hallfrisch J, Qiao N, Muller D, Andres R, Fleg JL, et al. The combination of high fruit and vegetable and low saturated fat intakes is more protective against mortality in aging men than is either alone : the Baltimore longitudinal study of aging. J Nutr. 2005;135(3):556–61. 10.1093/jn/135.3.556. 10.1093/jn/135.3.556 15735093

[75] Barker M, St-Onge M, Seixas A, Killgore WD, Wills CC, Grandner MA (2020). 0140 dietary macronutrients and sleep duration, sleep disturbance, and daytime fatigue. Sleep.

[76] St-Onge MP, Roberts A, Shechter A, Choudhury AR (2016). Fiber and saturated fat are associated with sleep arousals and slow wave sleep. J Clin Sleep Med.

[77] St-Onge MP, Crawford A, Aggarwal B (2018). Plant-based diets: reducing cardiovascular risk by improving sleep quality?. Curr Sleep Med Rep.

[78] Åkerstedt T, Ghilotti F, Grotta A, Bellavia A, Lagerros YT, Bellocco R (2017). Sleep duration, mortality and the influence of age. Eur J Epidemiol.

[79] Liu TZ, Xu C, Rota M, Cai H, Zhang C, Shi MJ, et al. Sleep duration and risk of all-cause mortality: a flexible, non-linear, meta-regression of 40 prospective cohort studies. Sleep Med Rev. 2017;32:28–36. 10.1016/j.smrv.2016.02.005. 10.1016/j.smrv.2016.02.005 27067616

[80] Yang L, Yang H, He M, Pan A, Li X, Min X, et al. Longer sleep duration and midday napping are associated with a higher risk of CHD incidence in middle-aged and older Chinese: the Dongfeng-Tongji cohort study. Sleep. 2016;39(3):645–52. 10.5665/sleep.5544. 10.5665/sleep.5544 PMC4763372 26564127

[81] Jiang CQ, Xu L, Lam TH, Jin YL, Sen Zhang W, Zhu F, et al. Glycemic measures and risk of mortality in older Chinese: the Guangzhou biobank cohort study. J Clin Endocrinol Metab. 2020;105(3):e181–90. 10.1210/clinem/dgz173. 10.1210/clinem/dgz173 31679008

[82] Liu Y, Zang Y, Yang Y (2020). China’s rural revitalization and development: theory, technology and management. J Geogr Sci.

