# Supplementary material for: Sleep duration, vegetable consumption and all-cause mortality among older adults in China: a 6-year prospective study
Source: BMC Geriatr. 2021 Jun 21;21:373. doi: 10.1186/s12877-021-02278-8 (PMC8215744; doi:10.1186/s12877-021-02278-8)
Supplement: Supplementary file 1 — Additional file 1. [file 12877_2021_2278_MOESM1_ESM.pdf]

Appendix

Table A1. Characteristics of study participants classified by sleep duration (weighted)

	Total	Hours of sleep per day					p-value
		≤5	6	7-8	9	≥10	
% of the weighted sample	NA	12.1	16.5	45.3	9.2	16.9	NA
**% Death in 2008-2014**	23.4	23.7	21.2	21.2	24.0	30.6	<0.001
**Frequency of vegetable consumption**							
% High	92.0	89.3	93.0	93.5	89.9	90.3	<0.001
**Demographics**							
Age (mean, years)	73.3	74.0	72.8	72.6	73.2	74.9	<0.001
% Aged over 80 years	17.2	19.9	15.3	14.3	17.3	25.1	<0.001
% Men	49.0	42.4	45.2	49.3	54.5	53.3	<0.001
% Urban	39.0	38.3	41.4	38.9	37.0	38.9	<0.001
**Socioeconomic factors**							
% 0 years of schooling	56.1	50.3	60.1	58.3	55.5	50.8	<0.001
% Economic independence	50.7	46.1	53.9	54.4	47.2	42.6	<0.001
% Professional occupation	12.6	10.8	14.0	14.0	10.6	9.8	<0.001
% Access to healthcare	93.6	88.7	92.5	94.5	95.8	94.6	<0.001
**Family/Social support**							
% Married	64.1	57.5	66.1	66.6	65.6	59.4	<0.001
% Close proximity to children	84.8	80.9	85.2	84.6	85.4	87.4	<0.001
**Health practice**							
% Doing regular exercise	37.7	40.8	39.4	39.1	35.5	31.2	<0.001
% Currently smoking	24.9	23.6	25.0	24.9	23.4	26.5	<0.001
**Health condition**							
% ADL disabled	4.7	6.0	4.5	3.0	2.9	9.3	<0.001
% Cognitively impaired	14.3	16.7	12.6	11.7	12.7	22.3	<0.001
% Self-reported poor health	49.1	65.7	56.5	45.7	42.9	45.2	<0.001

Note: (1) NA: not applicable. (2) Age was measured in mean, whereas all other variables were measured in percentage. (3) Except for the total sample whose weighted proportion was calculated across sleep hours, weighted percentages for all other variables were calculated within each sleep hours. (4) Except for the variable death, all other variables were measured at baseline wave in 2008. (5) p values were based on chi-square tests (for categorical variables) except for mean age that was derived from analysis of variance.

Table A2. Relative hazards of subsequent mortality by sleep duration (per day) and vegetable consumption, CLHLS, the 2008–2014 panel

	Model I	Model II	Model III	Model IV	Model V
**Sleeping hours/day**					
≤5 hours (7-8)	1.18***		1.15***	1.14***	1.08*
6 hours (7-8)	1.12***		1.11**	1.10**	1.06
9 hours (7-8)	1.03		1.04	1.04	1.04
≥10 hours (7-8)	1.27***		1.26***	1.25***	1.16***
**Frequency of vegetable consumption**					
High (low)		0.81***	0.83***	0.86***	0.94*
**Covariates**					
Demographics					
Age (mean, years)	1.08***	1.08***	1.07***	1.07***	1.06***
Men(women)	1.23***	1.23***	1.31***	1.41***	1.47***
Urban (rural)	0.92***	0.92***	0.97	1.01	0.95*
Socioeconomic factors					
1+ years of schooling (0)			0.97	1.00	1.05
Economic independence (no)			0.74***	0.80***	0.80***
Professional occupation (no)			1.11**	1.12**	1.09*
Access to healthcare (no)			0.85***	0.87***	0.92*
Family/Social support					
Currently married (no)				0.80***	0.81***
Close proximity to children (no)				1.04	1.02
Health practice					
Doing regular exercise (no)				0.92**	0.96
Currently smoking (no)				0.69***	0.78***
Health condition					
ADL disabled (no)					1.58***
Cognitively impaired (no)					1.45***
Self-reported good health (no)					1.15***
N (observations)	24,556	24,556	24,556	24,556	24,556
-log likelihood	19529.7***	19552.4***	19448.2***	19311.5***	18902.5***

Note: Relative hazards were obtained from parametric survival analyses that included those lost to follow-up based on imputed data. The category of each variable in the parentheses is the reference group of that variable. ***p<0.001, **p<0.01, *p<0.05, +p<0.1.

Table A3. Relative hazards of mortality for the interaction between sleeping hours (per day) and vegetable consumption, CLHLS, the 2008-2014 panel

	**Total**	**Women**	**Men**	**Young old**	**Oldest old**	**Rural**	**Urban**	**Illiterate**	**Literate**
**Panel A**									
≤5 hours and not often eating vegetables	1.08*	1.07	1.10+	0.89	1.08*	1.07	1.08	1.08+	1.08
6 hours and not often eating vegetables	1.06	1.07	1.05	0.95	1.07+	1.04	1.08	1.05	1.07
**7-8 hours and not often eating vegetables**	**1.00**	**1.00**	**1.00**	**1.00**	**1.00**	**1.00**	**1.00**	**1.00**	**1.00**
9 hours and not often eating vegetables	1.04	1.11+	0.97	0.80	1.07	1.04	0.97	1.08	0.96
≥10 hours and not often eating vegetables	1.16***	1.17***	1.15***	1.11	1.18***	1.15***	1.17***	1.17***	1.13*
High frequent intake of vegetables(low)	0.93*	0.98	0.88**	1.03	0.93*	0.97	0.89**	0.93*	0.96
**Panel B**									
≤5 hours and not often eating vegetables	1.30**	1.31**	1.31*	0.95	1.30**	1.33**	1.23	1.32**	1.21
6 hours and not often eating vegetables	1.02	1.02	1.02	1.26	1.01	1.00	1.05	1.09	0.81
**7-8 hours and not often eating vegetables**	**1.00**	**1.00**	**1.00**	**1.00**	**1.00**	**1.00**	**1.00**	**1.00**	**1.00**
9 hours and not often eating vegetables	0.93	0.89	0.98	0.92	0.92	0.89	0.99	0.98	0.78
≥10 hours and not often eating vegetables	1.30***	1.34***	1.23*	1.12	1.31***	1.19*	1.43***	1.29***	1.27+
≤5 hours and often eating vegetables	1.04	1.09	0.97	0.97	1.04	1.02	1.06	1.04	1.03
6 hours and often eating vegetables	1.07	1.15*	0.97	1.01	1.09	1.06	1.09	1.06	1.09
7-8 hours and often eating vegetables	1.01	1.07	0.92	1.10	1.01	1.01	1.00	1.02	0.98
9 hours and not eating vegetables	1.07	1.21*	0.89	0.86	1.09	1.14	0.97	1.11	0.96
≥10 hours and often eating vegetables	1.15*	1.21**	1.06	1.22	1.14*	1.16*	1.13	1.16*	1.08
**Panel C**									
≤5 hours and not often eating vegetables	1.29***	1.23**	1.42**	0.87	1.30**	1.32**	1.22+*	1.30**	1.24
6 hours and not often eating vegetables	1.02	0.97	1.11	1.13	1.00	0.99	1.06+	1.08	0.82
7-8 hours and not often eating vegetables	0.99	0.94	1.08	0.91	0.99	0.99	1.00	0.98	1.02
9 hours and not often eating vegetables	0.92	0.83	1.06	0.84	0.92	0.88	0.99	0.96	0.80
≥10 hours and not often eating vegetables	1.28***	1.25***	1.34***	1.02	1.30***	1.18**	1.43***	1.27***	1.29**
≤5 hours and often eating vegetables	1.03	1.02	1.05	0.89	1.03	1.01	1.06	1.02	1.06
6 hours and often eating vegetables	1.06+	1.08	1.05	0.92	1.08+	1.05	1.09	1.06	1.11+
**7-8 hours and often eating vegetables**	**1.00**	**1.00**	**1.00**	**1.00**	**1.00**	**1.00**	**1.00**	**1.00**	**1.00**
9 hours and not eating vegetables	1.06	1.14*	0.96	0.78	1.08+	1.12+	0.97	1.09+	0.99
≥10 hours and often eating vegetables	1.14***	1.14**	1.14**	1.11	1.14***	1.14***	1.13**	1.15*	1.10+
N (observations)	24,556	13,918	10,638	6,887	17,669	13,689	10,867	15,358	9,198

Note: Relative hazards were obtained from parametric survival analyses that included those lost to follow-up based on imputed data. The reference group in Panel B is "7-8 hours and not often eating vegetables", whereas the reference group in Panel C is "7-8 hours and often eating vegetables." All models controlled for all covariates in Table 1. Relative hazards for all covariates were not presented. ***p<0.001, **p<0.01, *p<0.05, +p<0.1.
